# FrenchFISH: Poisson Models for Quantifying DNA Copy Number From Fluorescence In Situ Hybridization of Tissue Sections

**DOI:** 10.1200/CCI.20.00075

**Published:** 2021-02-11

**Authors:** Geoff Macintyre, Anna M. Piskorz, Adam Berman, Edith Ross, David B. Morse, Ke Yuan, Darren Ennis, Jeremy A. Pike, Teodora Goranova, Iain A. McNeish, James D. Brenton, Florian Markowetz

**Affiliations:** ^1^ Cancer Research UK, Cambridge Institute, University of Cambridge, Cambridge, UK; ^2^ Cavendish Laboratory, Department of Physics, University of Cambridge, Cambridge, UK; ^3^ University of Glasgow, Glasgow, UK; ^4^ Institute of Cancer Sciences, University of Glasgow, Glasgow, UK; ^5^ Department of Surgery and Cancer, Imperial College London, UK; ^6^ Centre of Membrane Proteins and Receptors (COMPARE), Universities of Birmingham and Nottingham, UK

## Abstract

Chromosomal aberration and DNA copy number change are robust hallmarks of cancer. The gold standard for detecting copy number changes in tumor cells is fluorescence in situ hybridization (FISH) using locus-specific probes that are imaged as fluorescent spots. However, spot counting often does not perform well on solid tumor tissue sections due to partially represented or overlapping nuclei.

**MATERIALS AND METHODS:**

To overcome these challenges, we have developed a computational approach called FrenchFISH, which comprises a nuclear volume correction method coupled with two types of Poisson models: either a Poisson model for improved manual spot counting without the need for control probes or a homogeneous Poisson point process model for automated spot counting.

**RESULTS:**

We benchmarked the performance of FrenchFISH against previous approaches using a controlled simulation scenario and tested it experimentally in 12 ovarian carcinoma FFPE-tissue sections for copy number alterations at three loci (c-Myc, hTERC, and SE7). FrenchFISH outperformed standard spot counting with 74% of the automated counts having < 1 copy number difference from the manual counts and 17% having < 2 copy number differences, while taking less than one third of the time of manual counting.

**CONCLUSION:**

FrenchFISH is a general approach that can be used to enhance clinical diagnosis on sections of any tissue by both speeding up and improving the accuracy of spot count estimates.

## INTRODUCTION

Chromosomal instability coupled with defective DNA repair can cause loss or duplication of DNA, a characteristic attribute of cancer cells.^1^ Interrogation of DNA copy number aberrations is critical for diagnosis^2^ and understanding tumor etiology.^1^ Technologies for measuring DNA copy number have evolved from optical profiling of single loci^3^ through to sequencing of the entire tumor genome.^4^ However, determining the absolute number of copies from bulk sequencing data remains difficult because of normal cell contamination and intratumor heterogeneity,^5^ and the results are generally reported in terms of loss or gain of DNA relative to an assumed diploid or median estimate of ploidy. Information from methods that assay single loci is often required to validate these estimates of absolute copy number.^6,7^

**Context**

**Key Objective**
How can we account for noise and the overlap of nuclei when counting probes in images of tissue sections prepared with fluorescence in situ hybridization (FISH)?
**Knowledge Generated**
We developed FrenchFISH, a nuclear volume correction method, comprising two types of Poisson models: The first model improves manual spot counts without the need for control probes, and the second improves spot counts generated automatically by specialized computer vision software.
**Relevance**
Our work provides a method for improving the accuracy of FISH copy number estimates, thereby improving clinical outcomes relying on those data.

**FIG 1.**
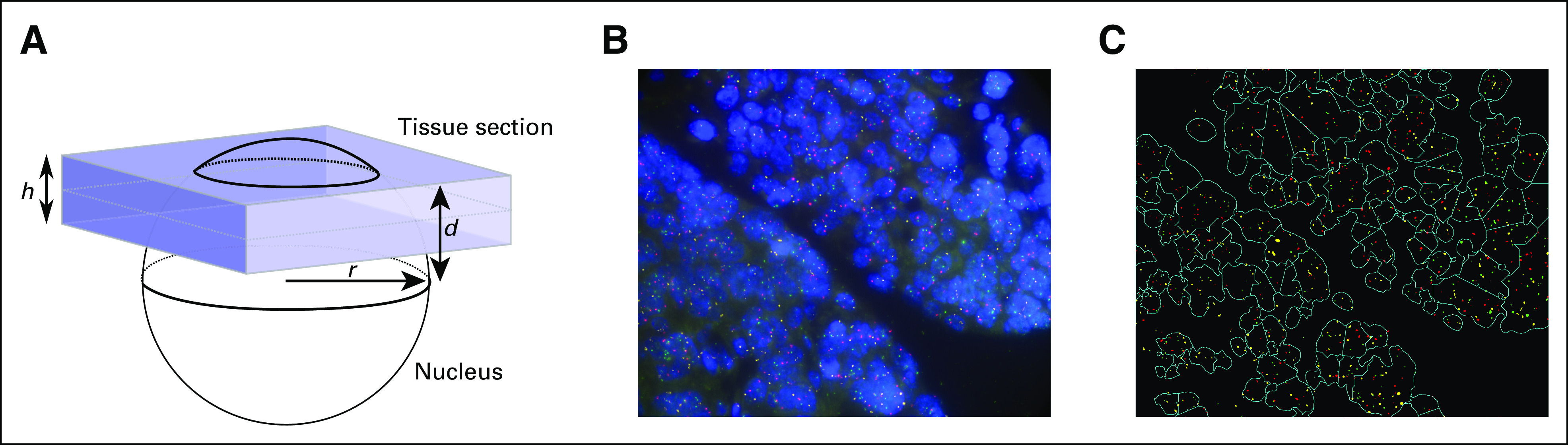
Tissue section FISH. (A) A schematic of a tissue section cutting through a cell nucleus. The highlighted quantities are used for calculating the volume of nucleus appearing in the tissue section. (B) Three probe fish were applied to high-grade serous ovarian carcinoma. (C) Automatic image segmentation and spot recognition applied to the image in B. Note the difficulty in accurately separating overlapping nuclei. FISH, fluorescence in situ hybridization.

Fluorescence in situ hybridization (FISH) of interphase nuclei is the most widely established technique for interrogating single locus copy number. Fluorescent probes are hybridized to a specific genomic region of interest and appear as discrete foci when visualized with fluorescent microscopy.^8^ The standard analysis of FISH data relies on time-consuming manual counting of spots in these images.^9^ Automated systems to quantify foci using nuclei recognition and spot counting algorithms (reviewed in ref. 10 ) aim to make the analysis of FISH data less labor-intensive, faster, and more objective. However, the accuracy of most systems is limited to identification of spots in intact and separated nuclei.^11^ These systems have high utility and accuracy for specimens from hematological malignancies as good cell separation can be achieved. However, diagnostic sections of solid tumor tissue pose a significant challenge for both automated and manual analyses. Accurate identification of single nuclei either by eye or by automatic image segmentation can be hard when nuclei cluster closely and overlap (Fig 1). Arbitrary cut points between grouped nuclei are typically used to separate these clusters, leading to noisy estimates of spot counts. Additionally, tissue sections are typically 3-5 µm, which is smaller than the diameter of most tumor nuclei, and thus, the majority of nuclei are not captured completely in the volume of the section.^12,13^

## MATERIALS AND METHODS

### Simulation

Simulated tissue sections were generated using the following procedure:

1. Fix tissue section height h at 3 µm and the nuclei radius r to 9 µm.
2. For C = 50 cells per simulated tissue section, estimate d (the distance from the midline of the nucleus to the top of the tissue section):

$${\text{d}}_{\text{c}}∼\text{Uniform}\left(0,\text{r}-\frac{2}{\text{h}}\right)$$

3.For each d_c_, calculate the fraction of the nucleus contained in the section,
  $${\text{V}}_{\text{frac}}\left({\text{d}}_{\text{c}}\right)=\frac{{\text{V}}_{\text{seg}}\left(9,{\text{d}}_{\text{c}}3\right)}{{\text{V}}_{\text{sphere}}\left(\text{r}\right)}$$
4.Using V_frac_ as the prior probability for seeing a spot sampled from a Poisson distribution, generate observed spot counts

$${\text{n}}_{\text{c}}^{\text{ctrl}}∼\text{Poisson}\left({\text{n}}_{\text{c}}^{\text{ctrl}}\times {\text{V}}_{\text{frac}}\right){\text{and n}}_{\text{c}}∼\text{Poisson}\left({\text{n}}_{\text{c}}\times {\text{V}}_{\text{frac}}\right),{\text{for n}}_{\text{c}}\neq {\text{n}}_{\text{c}}^{\text{ctrl}}\;$$ (1)

5.If the probability of overlap *P* is > 0, merge with neighboring nucleus c + 1, recalculating the overlapped nuclei area a:

$$\text{a}={\text{πr}}^{2}-2{\text{r}}^{2}\cos^{-1}\left(\frac{\text{b}}{2\text{r}}\right)-\frac{\text{d}}{2}\sqrt{4{\text{r}}^{2}-{\text{d}}^{2}}\;$$ (2)

where b is the distance between nuclei centrepoints sampled from d ∼ Uniform((0, 0.3]) and updated spot count:

$${\text{n}}_{\text{c}}={\text{n}}_{\text{c}}+{\text{n}}_{\text{c}+1}\;$$ (3)

6.If error e is ≠ 0, then update spot count:

$${\text{n}}_{\text{c}}=\{{\text{n}}_{\text{c}}+1\text{ if e}\geq 0\;{\text{n}}_{\text{c}}-1\text{ if e}\leq 0\;$$ (4)

7.Repeat the above steps 10 times for all possible combinations of e ∈ {−0.2, −0.1, −0.05, 0, 0.05, 1, 0.2}, p ∈ {0, 0.1, 0.3, 0.5, 0.8}, ${\text{n}}_{\text{c}}^{\text{ctrl}}$ ∈ {1, 2, 3, 4}, and n_c_ ∈ {1, 2, 3, 4, 5, 6, 7, 8, 9, 10}.

### FISH on ovarian carcinoma tissue sections

#### Patient Sample Selection.

Eight high-grade serous ovarian carcinoma samples were selected and reviewed by a pathologist who marked the area of each tumor on the H&E sections. In addition, four samples from two cases of ovarian squamous cell carcinoma arising in mature cystic teratoma were also selected.

Details of these cases (patients 7 and 11) have been published previously.^17^ All paraffin blocks were sectioned at 3 µm on positively charged microscope slides.

#### Fluorescent In Situ Hybridization.

FISH was performed on 3-µm tissue sections on positively charged slides using the probe cocktail composed of hTERC (3q26), c-Myc (8q24), and SE7 Triple color (KBI-10704, Leica Microsystem). Tissue digestion and probe hybridization was performed according to the manufacturer's recommendations using Poseidon Tissue Digestion Kit I (KBI-60007 Tissue Digestion Kit I, Leica Microsystem) with the following modifications: tissue was pretreated in solution A (LK-110B) at 96 °C to 98 °C for 10 minutes and digested using pepsin solution (LK-110B) for 5 minutes. FISH digital images were captured by using a Nikon Eclipse fluorescence inverted microscope equipped with a charge-coupled device camera (Andor Neo sCMOS), using filter sets for DAPI/YGFP/TRITC/CY GFP with an objective lens (Plan Apo VC 100×, Nikon). All images were captured with 100× magnification of the objective and a pixel size of 0.07 µm. For each selected field, 21 Z sections were taken with a step size of 0.3 µm. Large images of 7 × 7 fields were automatically captured from each tissue section, and the 5 best fields of view with adequate tumor tissue, free of optical artifact, were chosen for further analysis with the exception of JBLAB-178 where only 2 fields of view were suitable.

#### Image Processing.

FISH of tissue sections are noisy and display a number of recurring artifacts, which can be mitigated using image preprocessing methods. The main artifacts are bright error spots outside the nucleus that reduce true spot signal, precipitation that causes faint, erroneous spots within the nucleus, and autofluorescence of areas outside the nucleus. To overcome these issues, the following procedure was followed, which forms part of FrenchFISH image preprocessing:

1. Using Fiji:
  • for each field of view, the position in the z-stack with the best focus was detected using Vollath's F_4_ measure.^18^
  • The 4 stacks below and 5 stacks above were retained.
  • A maximum intensity projection was taken across the stacks to generate a single image for further processing.
  • The contrast of each spot channel was normalized and adjusted, allowing a saturation of up to 40% of the image. This allowed the weaker spot signals to be matched to the stronger, extranuclear noise spots.
2. Using R:
  • Nuclear staining is segmented using the FISHalyzer^19^ package.
  • Spot channel images are masked using nuclear segmentation.
  • The image is filtered and normalized retaining on the top 10% of signal intensity to remove remaining autofluorescence.
  • A two-stage Gaussian blurring and automatic thresholding approach is applied using the Intermodes^20^ method for channels with a precipitation signal, and the Renyi Entropy^21^ method for those without precipitation, found in the autothresholdr package.^22^ This combines and removes any small spot artifacts.
  • A size-based filter is applied for final spot segmentation.

#### Manual Spot Counting.

Manual spot counting was performed using IMARIS8 software following this procedure:

- Import nd2 image (21 z-stacks).
- Display in 3D.
- Display DAPI channel and switch off all other channels.
- Print image.
- Identify nuclei suitable for manual spot counting (none or minimal nuclear overlap cell nuclei for signal counting), circle them on the 2D image on the paper, and give them numbers. Move the 3D image around to see if the nuclei are nicely separated.
- Set up an aqua channel so that artifacts are removed and dots are clearly visible.
- Set up a red channel so that artifacts are removed and dots are clearly visible.
- Set up a green channel so that artifacts are removed and dots are clearly visible.
- For every selected nucleus, perform the following steps:
  - Measure the size of the nuclei (x- and y-plane diameters).
  - Count spots in the aqua channel and record.
  - Count spots in the red channel and record.
  - Count spots in the green channel and record.

To address these challenges, both manual and automated analyses have been improved by using control probes that bind to a specific locus with the known copy number state n_ctrl_.^10^ Two commonly used approaches are as follows:

1. Only nuclei containing the expected number of control probes (usually n_ctrl_ = 2) are used to estimate the copy number of other loci. The underlying assumption is that if a nucleus contains the expected number of control probes, then it is likely that the majority of the nucleus is captured by the section, and hence, other spots will be well-represented.
2. The spot count for the locus of interest is scaled by the ratio of expected over observed control probe copy number:

$$\text{n}=\frac{{\text{n}}_{\text{ctrl}}^{\text{exp}}}{{\text{n}}_{\text{ctrl}}^{\text{obs}}}\times {\text{n}}^{\text{obs}}\;$$ (5)

In this case, the underlying assumption is that the number of observed control spots is linearly correlated with the number of spots observed for the locus of interest.

However, there are significant limitations associated with both of these methods. For example, a 3-µm-thick tissue section containing cells with a nuclear diameter of 9 µm will, on average, have only 41% of each nucleus represented in the section (Fig 1A). Therefore, for method 1, it is unlikely that the section will contain many nuclei with a complete control probe count, and the locus of interest is likely to be undersampled. Using thicker tissue sections can overcome this limitation; however, as the thickness of the section increases, the image quality decreases, and many more overlapping nuclei are captured, which further complicates identification of single nuclei. Method 2 performs well when the control probe is at the expected copy number. However, in tumors with significant aneuploidy, it is difficult to identify a control probe with constant copy number, even when using centromeric probes.

Thus, new automated approaches are required that generate robust and reproducible results from fixed tumor sections. Ideally, new methods should account for the three major challenges in FISH analysis of tissue sections: (1) nucleus subsampling, (2) control probe aneuploidy, and (3) overlapping nuclei. We have addressed these challenges by developing FrenchFISH, a computational package that comprises three major computational innovations for improved spot counting: volume adjusted spot counting, which accounts for partial nucleus representation without the need for control probes; Poisson estimated spot counts from manually counted nuclei, which account for uncertainty in spot counts; and a homogeneous Poisson point process model, which facilitates automated spot counting and circumvents the need for single nucleus image segmentation. Here, we show the derivation of the FrenchFISH model and show that it outperforms standard spot counting approaches and is significantly faster than manual spot counting.

## RESULTS

### The FrenchFISH Model

The goal of the analysis is to estimate the copy number of a locus denoted by n, which we will achieve by volume-adjusting observed spot counts and using Poisson models.

#### Observed spot counts.

FISH of a probe specific to the locus allows us to observe copy number in terms of spot counts inside the nucleus. Here, we assume that a FISH image has C ∈ N = {1, 2, …} cell nuclei and the number of observed spots in cell c ∈ [C] = {1, …, C} is n^obs^. The average number of observed copies of the locus in the tissue section is

$${\text{n}}^{\text{obs}}=\frac{1}{\text{C}}\sum_{\text{c}=1}^{\text{C}}{\text{n}}_{\text{c}}^{\text{obs}}\;$$ (6)

#### Volume adjusted spot counting.

Figure 1A displays a schematic of a nucleus subsampled as a result of tissue section cutting. For simplicity, we assume that all nuclei are spherical with radius r (r is typically estimated from image). Their volume is calculated by

$${\text{V}}_{\text{sphere}}\left(\text{r}\right)=\frac{4}{3}\pi {\text{r}}^{3}\;$$ (7)

For a specified section thickness h, we can express the volume of the nucleus sampled by a section in terms of d, the distance of the section edge from the nucleus midline:

$${\text{V}}_{\text{seg}}\left(\text{d}\right)=\pi \text{h}\left({\text{r}}^{2}-{\text{d}}^{2}-\text{hd}-\frac{1}{3}{\text{h}}^{2}\right)\;$$ (8)

By integrating over d and dividing by h, we can compute the average volume sampled:

$${\text{V}}_{\text{avg}}=\frac{1}{\text{h}}-\int_{\text{0}}^{\text{h}}\text{Vseg d}d=\pi \text{h}\left(\frac{2}{3}{\text{r}}^{2}+\frac{\text{rh}}{3}-\frac{{\text{h}}^{2}}{6}\right)\;$$ (9)

This quantity can be used to scale the observed number of spots to get an estimate of the true number of spots:

$${\text{n}}^{\text{voladj}}=\frac{{\text{V}}_{\text{sphere}}}{{\text{V}}_{\text{avg}}}\times {\text{n}}^{\text{obs}}\;$$ (10)

#### Modeling uncertainty in manual spot counts.

As the observed spot counts are subject to both hybridization and image signal processing noise, we use a probabilistic model that accounts for this uncertainty. We model the counts as coming from a Poisson distribution with rate λ. Given this, the likelihood of our data can be expressed as

$$\text{P}\left({\text{n}}^{\text{voladj}}|\text{λ}\right)=\prod_{\text{c}=1}^{\text{C}}\frac{e^{-\text{λ}}{\text{λ}}^{{\text{n}}_{c}^{\text{voladj}}}}{{\text{n}}_{\text{c}}^{\text{voladj}}!}\;$$ (11)

To compute the posterior of λ given the data, we use Bayes' rule to transform the likelihood into

$$\text{P}\left(\text{λ}|{\text{n}}^{\text{voladj}}\right)=\text{P}\left({\text{n}}^{\text{voladj}}|\text{λ}\right)\cdot \text{P}\left(\text{λ}\right)\;$$ (12)

Using the conjugate gamma prior as P(λ) and the likelihood of Eq. 11, we sample from the posterior with Markov Chain Monte Carlo (MCMC) to generate λ_t_ ∈ [T] values fit to the data after a burn-in of 1,000 iterations. We use the MCpoissongamma function from the MCMCpack package^14^ in R to achieve this. From this sampling chain, we then compute the expected rate that is equal to the expected spot count:

$$\text{E}\left[\text{n}\right]=\text{E}\left[\text{λ}\right]=\frac{1}{\text{T}}\cdot \sum_{\text{t}=1}^{\text{T}}{\text{λ}}_{\text{t}}\;$$ (13)

#### Modeling uncertainty in automatic nuclear segmentation.

Although segmentation of single nuclei in tumor sections is difficult, separating nuclear staining from background and accurately defining spots remains relatively easy. Our approach exploits this fact in the framework of a homogeneous Poisson point process. A Poisson process models a continuous series of events across space or time. In our setting, we consider spots as events and nuclear area a measured in µm^2^ as space. The number of spots in an area a is denoted by N(a) and modeled by a Poisson process with intensity λ^PP^:

$$\text{P}\left(\text{N}\left(\text{a}\right)=\text{n}\right)=\frac{1}{\text{n}!}{\left({\text{λ}}^{\text{PP}}\text{a}\right)}^{n}{\text{e}}^{-{\text{λ}}^{\text{PP}}}\text{a}\;$$ (14)

and using the fitPP.fun from the NHPoisson package^15^ in R, we obtain a maximum likelihood estimate for λ^PP^.

As λ^PP^ is a spot count estimate per µm^2^ of an observed nuclear area, to get the estimated number of spots per nucleus, we first multiply by the average area of a nucleus, πr^2^, and then scale by the average nuclear volume represented in the tissue section to get an estimate of the number of copies n:

$$\text{E}\left[\text{n}\right]={\text{λ}}^{\text{PP}}\times {\text{πr}}^{2}\times \frac{{\text{V}}_{\text{sphere}}}{{\text{V}}_{\text{avg}}}\;$$ (15)

### Validation and Benchmarking of FrenchFISH

To validate and benchmark FrenchFISH, we used the controlled scenario of a simulation study as well as a real-world case study in ovarian carcinoma.

#### Benchmarking in simulation study.

We simulated a total of 11,200 tissue sections to benchmark our approach. For each condition, we simulated 10 replicate sections with 50 nuclei. All nuclei had their midpoint location randomly positioned within the tissue section. Test conditions were selected from all possible combinations of the following:

- control probe copy number n_control_ ∈ {1, 2, 3, 4},
- probe of interest copy number n ∈ {1, 2, 3, 4, 5, 6, 7, 8, 9, 10},
- percentage of nuclei with a probe sampling error of plus or minus one count e ∈ {−20, −10, −5, 0, 5, 10, 20}, and
- probability of nucleus overlapping with another nucleus in the section *P* ∈ {0, 10, 30, 50, 80}.

Using these data, we tested the performance of FrenchFISH against the standard approach outlined in Eq. 5 where a control probe (assumed to be diploid) is used to scale the observed spot counts.

#### Benchmarking against noisy spot counts.

We first measured performance using simulated tissue sections with nonoverlapping nuclei and varying levels of noise. Noise was introduced by either undercounting or overcounting by one spot in 5%, 10%, or 20% of the cells in each tissue section.

**FIG 2.**
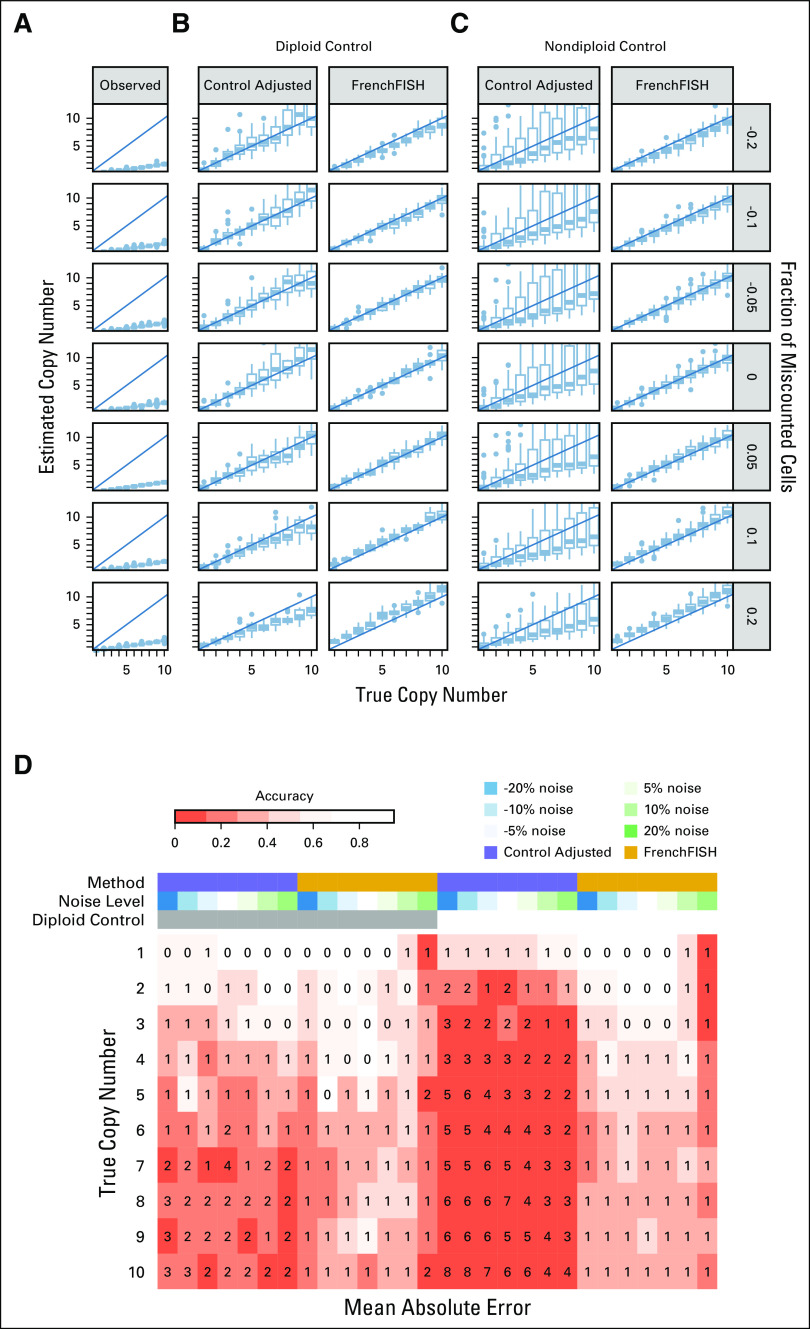
Performance assessment on simulated spot counts with varying levels of noise. (A) Box plots showing the distribution of unadjusted observed spot counts (*y*-axis) compared with the true spot counts (*x*-axis), for varying noise levels (y facets). (B) Spot count estimates for the standard control probe adjusted method (ControlAdjusted) and FrenchFISH. All simulated tissue sections in these plots had an accompanying diploid control probe count. (C) Spot count estimates for tissue sections with nondiploid controls. (D) A heatmap showing the accuracy of spot counting (shading) for each method, noise level, and control probe count. The integers inside the heatmap boxes show the mean absolute error. FISH, fluorescence in situ hybridization.

Naive spot counting without correction showed a severe underestimate of the true number of spots (Fig 2A). The standard correction approach improved spot count estimates when the control probe was diploid (Fig 2B). However, estimates showed high variability as the true number of spots increased. By contrast, FrenchFISH showed consistent performance across all true copy number states. Performance remained adequate for noise levels up to 10%. The standard approach showed worse performance than FrenchFISH at 20% noise; however, errors were less pronounced for undercounting noise compared with overcounting noise (Fig 2B). The standard approach largely failed to provide correct copy number estimates when the control probe copy number was other than diploid, especially for higher noise levels (Fig 2C). FrenchFISH did not show a deterioration in performance as it did not rely on a control probe.

To gain further insight, we observed accuracy and mean absolute error for both approaches under the same varying noise conditions (Fig 2D). Overall accuracy was poor for the standard approach except when the control probe was diploid and true copy number was 1. High accuracy was observed for FrenchFISH up to a true spot count of 4 and noise levels of 10%. Accuracy was poor in cases where overcounting noise was 20%. Despite a deterioration in accuracy beyond true copy number counts of 4, the mean absolute error for FrenchFISH never exceeded 1; thus, the FrenchFISH estimates were only ever wrong by one copy. By contrast, the standard approach had a mean absolute error of up to 7 under some conditions.

#### Benchmarking against overlapping nuclei.

**FIG 3.**
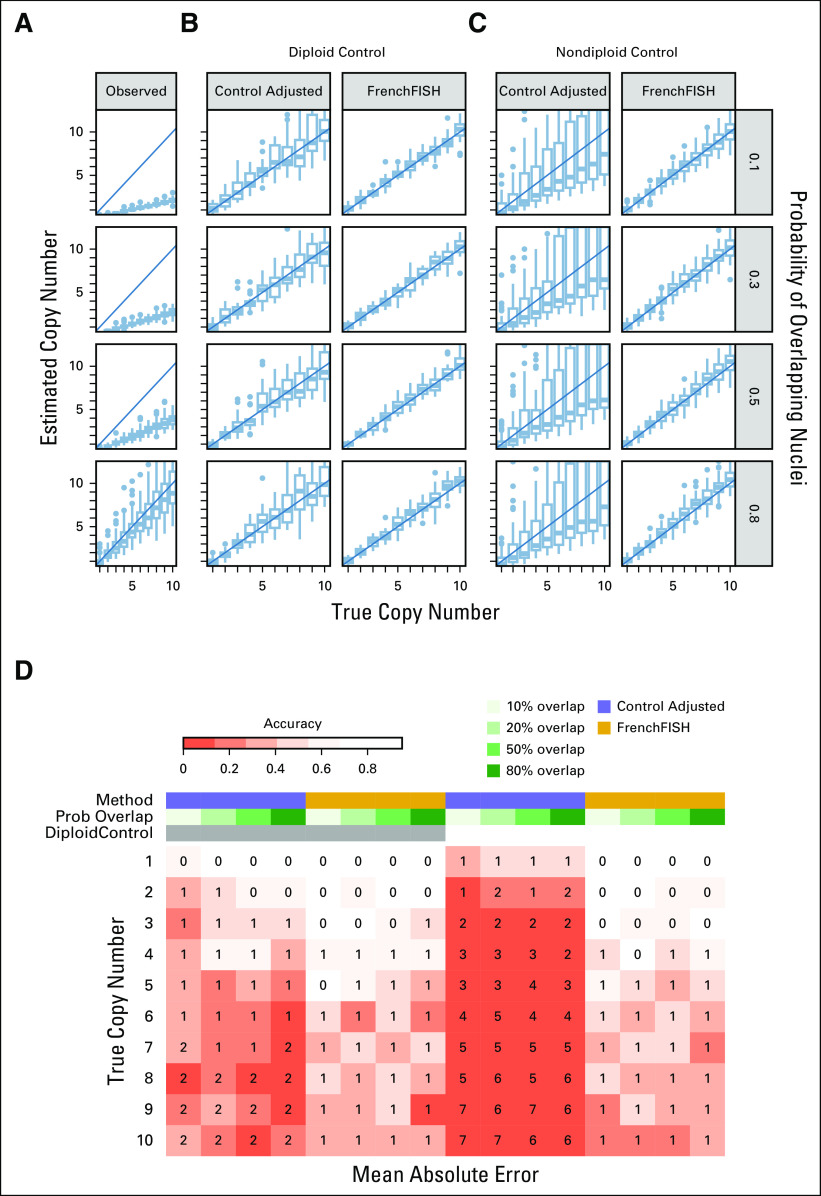
Performance assessment on simulated spot counts with varying levels of overlapping nuclei. (A) Box plots showing the distribution of unadjusted observed spot counts (*y*-axis) compared with the true spot counts (*x*-axis), for varying levels of probability of overlapping nuclei (y facets). (B) Spot count estimates for the standard control probe adjusted method (ControlAdjusted) and FrenchFISH. All simulated tissue sections in these plots had an accompanying diploid control probe count. (C) Spot count estimates for tissue sections with nondiploid controls. (D) A heatmap showing the accuracy of spot counting (shading) for each method, overlap probability, and control probe count. The integers inside the heatmap boxes show the mean absolute error. FISH, fluorescence in situ hybridization.

Here, we assessed the performance of both methods across simulated tissue sections with varying degrees of nuclear overlap (Fig 3). Both methods were robust to nuclear overlap in the diploid control probe setting, including at 80% probability of overlap. However, the standard approach again showed more variable results as the true copy number increased (Fig 3B). The standard approach consistently failed to estimate the correct copy number when the control probe was not diploid; however, this error did not vary with the degree of overlap (Fig 3C). FrenchFISH showed a mean absolute error no greater than one, whereas the standard approach showed up to two copies in the diploid control probe setting and up to seven copies in the nondiploid setting (Fig 3D).

#### Benchmarking against heterogeneous true copy number.

**FIG 4.**
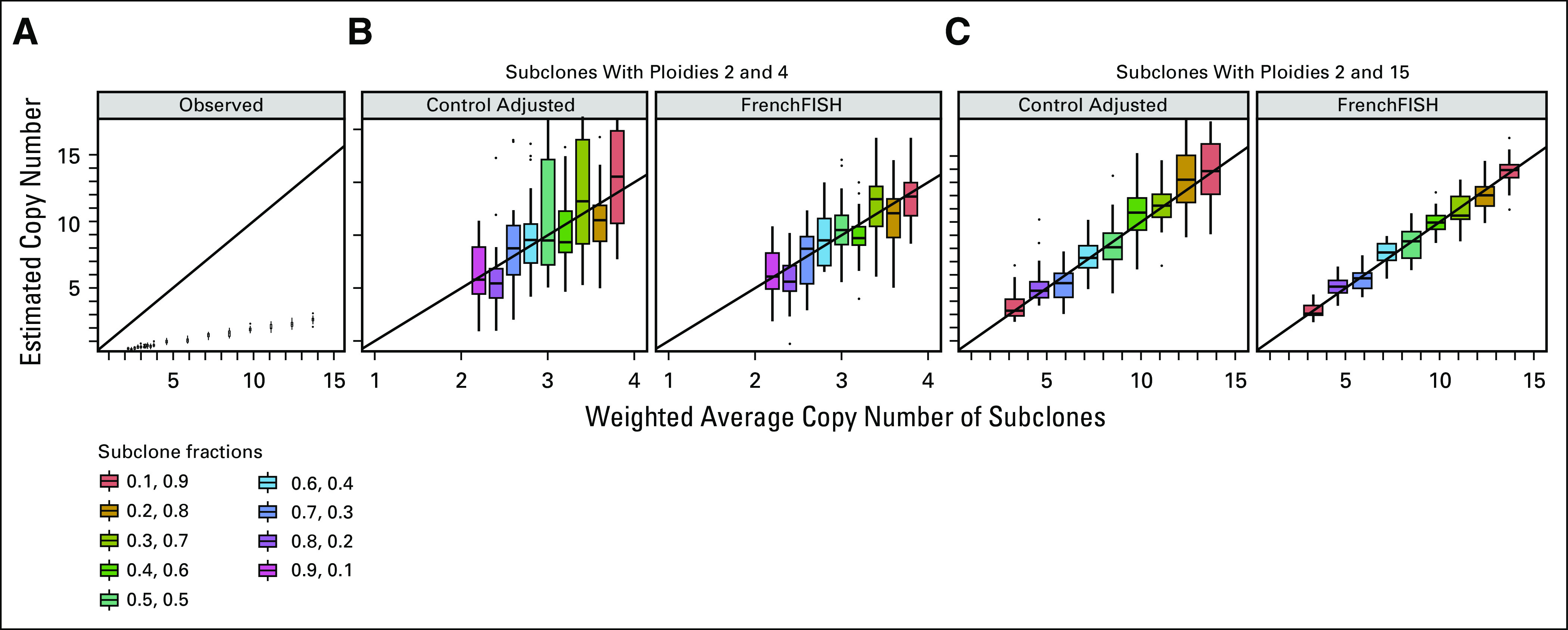
Performance assessment on spot counts in simulated sample with subclones of different ploidies. (A) Box plots showing the distribution of unadjusted observed spot counts (*y*-axis) compared with the true spot counts (*x*-axis) for a simulated tissue section containing varying subclonal mixtures (y facets). (B) Spot count estimates for the standard control probe adjusted method (ControlAdjusted) and FrenchFISH for a tissue section sample containing two subclones of ploidies 2 and 4 of varying fractional mixtures in the sample. Each distinct fractional mixture of subclones leads to a different weighted average ploidy of the whole sample (*x*-axis). (C) Spot count estimates for a simulated sample containing two subclones of ploidies 2 and 15. FISH, fluorescence in situ hybridization.

FrenchFISH relies on a homogeneous Poisson point process, which is an unrealistic assumption if multiple subclones with different ploidies are present in one image. To test how robust FrenchFISH is to subclonality, we simulated samples containing subclones of different ploidies and compared the FrenchFISH estimations on these simulations with the weighted average copy numbers of the whole samples. Samples containing a mixture of cells of ploidies 2 and 4 (Fig 4B), as well as samples with ploidies 2 and 15, were simulated (Fig 4C). Ten different fractions of each ploidy were simulated for each of these two mixtures. FrenchFISH performed consistently well at estimating the weighted average copy number across all twenty different bimodal subclonal simulations tested (4). This shows that FrenchFISH is robust to copy number heterogeneity.

#### Case study on ovarian carcinoma tissue sections.

We performed both manual and automatic spot counting on multichannel FISH of tissue sections from 12 ovarian carcinoma cases. Manual spot counts were corrected using the volume adjustment method in FrenchFISH, and automatic counting was performed using the Poisson point process model.

#### Manual versus automatic counting.

**FIG 5.**
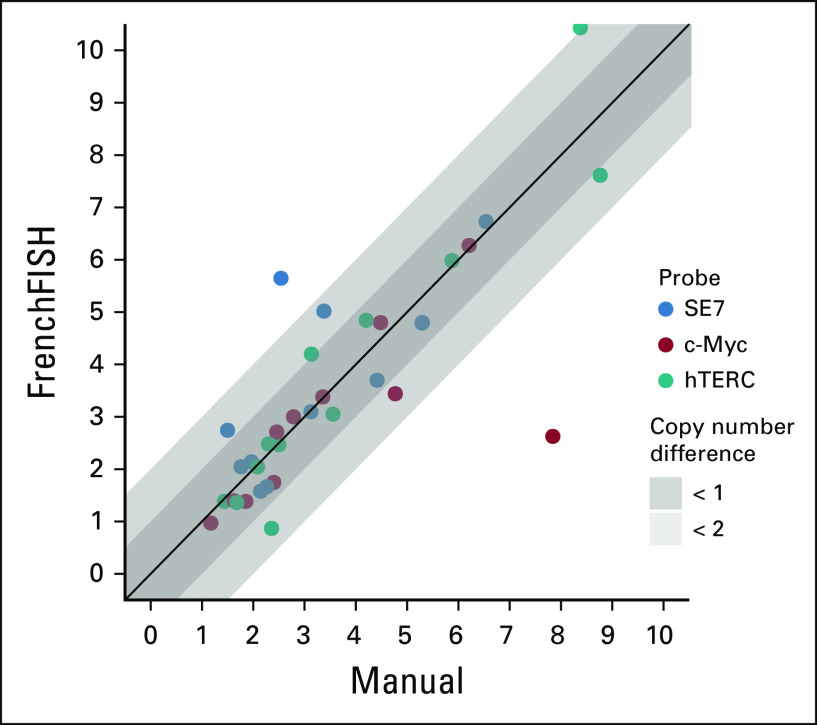
Spot count estimates for 3 probes across 12 ovarian carcinoma cases. This scatterplot shows spot count estimates from manual counting versus FrenchFISH automated spot counting. Points falling within the dark shaded area have estimates within 1 copy of each other across the methods. Those falling within the light gray area are within 2 copies. Those falling outside this area are greater than two copies different. FISH, fluorescence in situ hybridization.

We observed the degree of agreement between manual and automatic spot counting to assess whether the automatic method resulted in any loss of performance compared with manual counting. About 74% (26 of 35) of the estimated copy number counts were less than one copy number different with a further 17% (6 of 35) having estimates less than two copies different (Fig 5).

#### Timing analysis.

**FIG 6.**
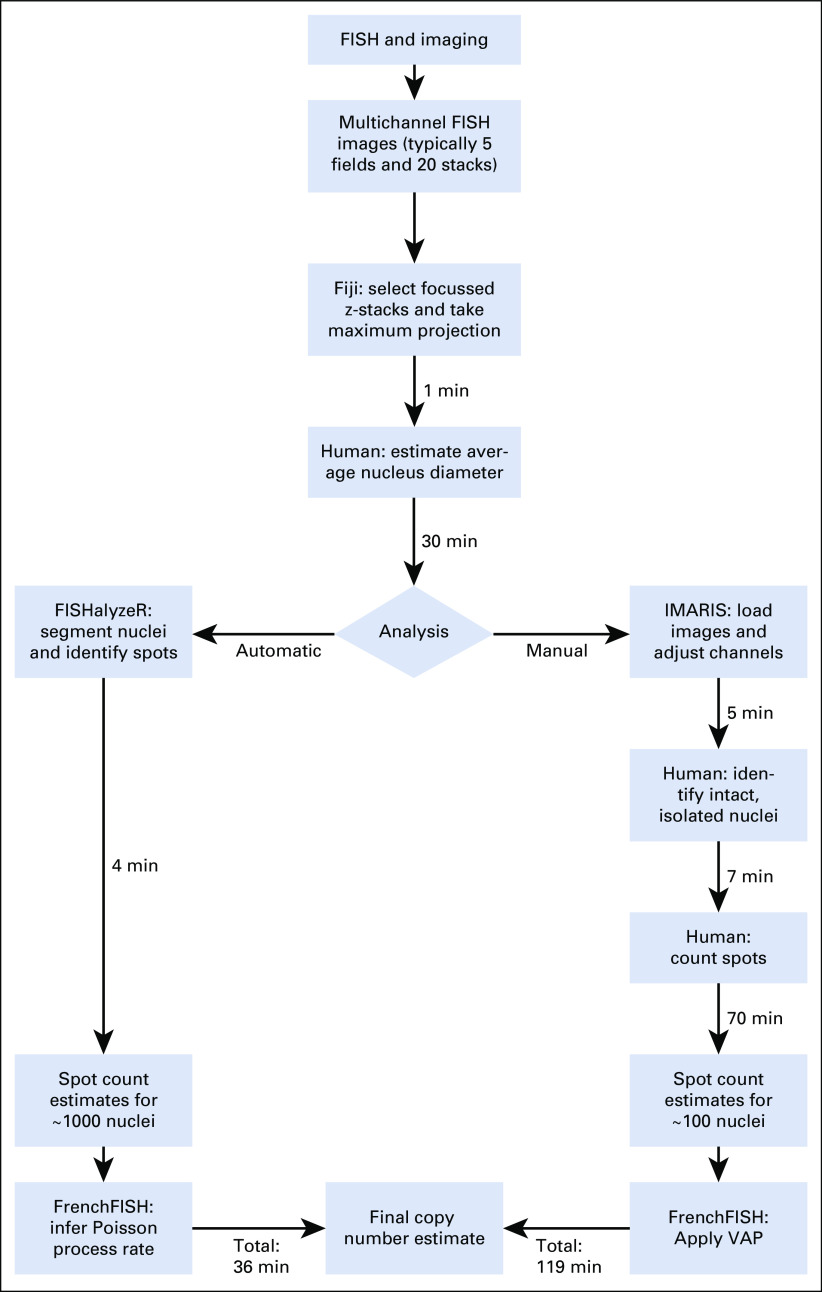
Comparison of manual spot counting and FrenchFISH automated spot counting, across 3 probes, using FISH of 12 ovarian carcinoma cases. This flowchart outlines the tasks required to carry out automatic or manual spot counting for a single sample. The minutes associated with each process are an average across 8 cases for up to 5 fields of view. Squares represent processes, the diamond represents a decision point, and the trapezoids represent input and/or output. For each process, it is listed whether it is carried out by software or by human. FISH, fluorescence in situ hybridization.

We measured the time it took to perform both manual and automatic spot counting. Figure 6 provides a breakdown of the two approaches and the timings associated with each step. Using up to 5 fields of view per sample, we were able to obtain roughly 100 manually curated nuclei per sample. The total average processing time for the automatic FrenchFISH approach was 36 minutes: 30 minutes for manual estimation of the nuclear diameter and then 6 minutes for software processing. The total average processing time for the manual approach was 119 minutes, with the majority of processing performed by a human.

## DISCUSSION

Here, we present FrenchFISH, a software tool for quantitative copy number estimation from FISH of tissue sections. FrenchFISH is the first method specifically designed to provide quantitative copy number estimates from tissue section FISH without the need for a matched control probe. FrenchFISH does not require time-intensive training and is quick to run because it derives its estimates from a Poisson point process.

We demonstrated the robust and superior performance of FrenchFISH using simulated tissue sections and FISH of ovarian carcinoma tissue sections. We explored the limitations of FrenchFISH using simulations of tissue sections with spot counting noise and overlapping nuclei. FrenchFISH was robust to overlapping nuclei noise and performed well in cases with up to 10% spot counting noise. Interestingly, overcounting noise resulted in worse performance than undercounting, suggesting that a conservative spot counting strategy could improve copy number estimates. Our controlled simulations also highlighted the difficulty in estimating high copy number states, with accuracy rapidly decreasing with copy numbers > 4 copies. However, in all cases tested, FrenchFISH estimates were not more than 1 copy different from the underlying truth. On ovarian carcinoma tissue sections, 74% of FrenchFISH automated spot count estimates were within 1 copy of manually counted estimates. This demonstrates that FrenchFISH is a viable alternative to manual counting, which would decrease analysis time fourfold with significantly less human intervention.

FrenchFISH also has some limitations. For instance, estimating the copy number of a sample from a limited number of FISH images presents an unavoidable sampling bias. Additionally, FrenchFISH relies on clearly defined and stained nuclear areas. If nuclear areas are too fuzzy, FrenchFISH will not be able to provide a reliable estimate. For example, the two spot count estimates that showed the largest difference compared with manual counting in Figure 5 can both be accounted for by probe or image artifacts: The image in which FrenchFISH overestimated the SE7 probe contained small spots on the image that exceeded the minimum detection threshold. Similarly, the c-Myc probe that was heavily underestimated was part of an image that contained an artifactual saturation of the probe signal outside of cell nuclei, which reduced the signal of the probes inside the cell.

Further validation of FrenchFISH is required using other complex data sets. Even with the advent of whole-genome sequencing (WGS) technologies to estimate copy number, there will always be a place for FISH to serve as a tool to validate WGS estimates in the clinic. For example, gains of the *ALK* locus have been identified as one of the original events in non–small-cell lung cancer.^16^ Since 10 or more copies of *ALK* distinguish this event from polysomy,^16^ FrenchFISH's ability to estimate precise copy number for solid tumor sections gives it unique utility in this and similar clinical situations.

FrenchFISH is implemented in R^23^ using elements of the FishalyzeR^19^ package. The FrenchFISH method has been published as an R package in Bioconductor,^24^ and instructions for its installation and use can be found in the repository given in the references.^25^ Code to reproduce all the results in this publication can be found in the FrenchFISH analyses repository.^26^ The ovarian carcinoma FISH images used in this study can be downloaded from the Cell Image Library.^27^
